# Sucrose synthase activity is not required for cellulose biosynthesis in Arabidopsis

**DOI:** 10.1111/tpj.15752

**Published:** 2022-04-21

**Authors:** Wei Wang, Sonja Viljamaa, Ondrej Hodek, Thomas Moritz, Totte Niittylä

**Affiliations:** ^1^ Department of Forest Genetics and Plant Physiology, Umeå Plant Science Centre Swedish University of Agricultural Sciences SE 901 83 Umeå Sweden; ^2^ Faculty of Health and Medical Sciences, Novo Nordisk Foundation Center for Basic Metabolic Research University of Copenhagen Copenhagen Denmark

**Keywords:** UDP‐glucose, cellulose, sucrose synthase, *Arabidopsis thaliana*

## Abstract

Biosynthesis of plant cell walls requires UDP‐glucose as the substrate for cellulose biosynthesis, and as an intermediate for the synthesis of other matrix polysaccharides. The sucrose cleaving enzyme sucrose synthase (SUS) is thought to have a central role in UDP‐glucose biosynthesis, and a long‐held and much debated hypothesis postulates that SUS is required to supply UDP‐glucose to cellulose biosynthesis. To investigate the role of SUS in cellulose biosynthesis of *Arabidopsis thaliana* we characterized mutants in which four or all six Arabidopsis *SUS* genes were disrupted. These *sus* mutants showed no growth phenotypes, vascular tissue cell wall defects, or changes in cellulose content. Moreover, the UDP‐glucose content of rosette leaves of the sextuple *sus* mutants was increased by approximately 20% compared with wild type. It can thus be concluded that cellulose biosynthesis is able to employ alternative UDP‐glucose biosynthesis pathway(s), and thereby the model of SUS requirements for cellulose biosynthesis in Arabidopsis can be refuted.

## Significance Statement

Many models of cellulose biosynthesis in plants imply that sucrose synthase channels the substrate UDP‐glucose to the plasma membrane localized cellulose synthase complex. Here we used sextuple Arabidopsis mutants devoid of sucrose synthase activity to test this hypothesis directly. The results show that sucrose synthase is not required for cellulose biosynthesis in Arabidopsis.

## INTRODUCTION

Cellulose, the main component of plant cell walls, is synthesized at the plasma membrane by the heteromeric cellulose synthase (cesA) rosette complex, which uses UDP‐glucose as a substrate (McFarlane et al., 2014). Early evidence from cotton fibers pointed to an important role for sucrose synthase (SUS) in sucrose cleavage and UDP‐glucose supply to cellulose biosynthesis. Significant proportion of SUS in the cotton fibers was associated with the membrane fraction, and *in situ* immunolocalization suggested SUS to be localized in the plasma membrane along the orientation of cellulose microfibrils (Amor et al., 1995; Haigler et al., 2001). These observations gave rise to a popular model, which depicts SUS in association with the cesA complexes channeling UDP‐glucose for cellulose biosynthesis, albeit sometimes SUS is drawn with an associated question mark to indicate uncertainty about the direct association (Endler & Persson, 2011; Guerriero et al., 2010; Haigler et al., 2001; McFarlane et al. 2014; Stein & Granot, 2019).

Observations in different plant species have supported the association of SUS and cesA complexes. A SUS antibody labeled reconstituted azuki bean (*Vigna angularis*) cesA complexes in an immunogold labeling assay, and it was reported that this SUS association with the rosette‐like structures was required for *in vitro* cellulose biosynthesis activity (Fujii et al., 2010). Cotton plants with suppressed SUS activity exhibited reduced initiation and elongation of the cellulose‐rich seed fibers, also supporting a SUS function in cellulose biosynthesis (Ruan et al., 2003). Immunoprecipitation of cesA complexes from developing wood of *Populus deltoides* × *canadensis* hybrid identified two SUS isoforms further suggesting a direct interaction between cesAs and SUS also in this species (Song et al., 2010).

Arabidopsis genome contains six *SUS* genes. Evidence challenging the role of SUS in cellulose biosynthesis was obtained by Barratt et al. (2009), who showed that the cellulose content in stems of quadruple Arabidopsis *sus1sus2sus3sus4* (*sus* ^*quad*^) mutants did not differ from the wild type. However, this conclusion was later questioned by Baroja‐Fernandez et al. (2012) who maintained that the quadruple Arabidopsis *sus* mutant contained sufficient SUS activity from the remaining two SUS isoforms SUS5 and SUS6 to support cellulose biosynthesis. Thus, the importance of SUS in providing UDP‐glucose to the CesA complexes is yet to be tested unequivocally. To settle the debate about SUS activity in the quadruple *sus* mutant we recently generated lines where all six Arabidopsis *SUS* genes were disrupted (Fünfgeld et al., 2022). The Fünfgeld et al. (2022) study focused on addressing the role of SUS in ADP‐glucose and starch biosynthesis, while here we have used the same *sus* mutants to assess SUS function in UDP‐glucose and cellulose biosynthesis.

## RESULTS AND DISCUSSION

**Figure 1:**
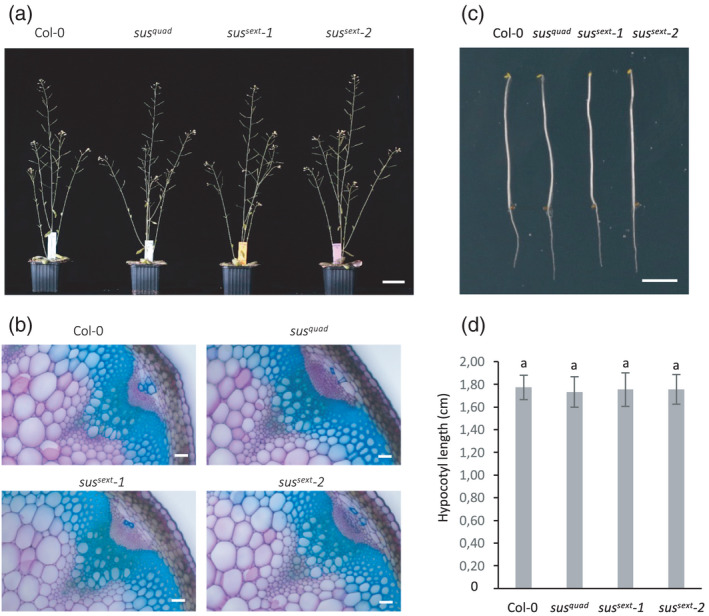
Phenotype of Arabidopsis *sus* mutants. (a) Eight‐week‐old wild type (Col‐0) and *sus* ^*quad*^, *sus* ^*sext*^ *‐1*, and *sus* ^*sext*^ *‐2* grown in long days (16‐h light, 8‐h dark). (b) Stem cross‐sections of Col‐0 and *sus* ^*quad*^, *sus* ^*sext*^ *‐1*, and *sus* ^*sext*^ *‐2* stained with 0.1% toluidine blue. Scale bars = 50 μm. (c) Etiolated Col‐0 and *sus* ^*quad*^, *sus* ^*sext*^ *‐1*, and *sus* ^*sext*^ *‐2* seedlings grown for 5 days in the dark. Scale bar = 0.5 cm. (d) Hypocotyl length of Col‐0 and *sus* ^*quad*^, *sus* ^*sext*^ *‐1*, and *sus* ^*sext*^ *‐2* seedlings grown for 5 days in the dark. Values are means ± SE, *n* = 25 biological replicates. One‐way ANOVA, *post‐hoc* Tukey HSD test. Letters indicate no significant differences (*P* < 0.05). [Colour figure can be viewed at wileyonlinelibrary.com]

To address the role of SUS activity in cellulose biosynthesis in Arabidopsis we made use of the previously generated quadruple and sextuple Arabidopsis *sus* mutants (Barratt et al., 2009; Fünfgeld et al., 2022). Readers are referred to Fünfgeld et al. (2022) for detailed description of the genotypes and the results showing that the two allelic *sus* ^*sext*^ *‐1* and *sus* ^*sext*^ *‐2* mutants contain no measurable SUS activity. Here, wild‐type, *sus* ^*quad*^ and the *sus* ^*sext*^ *‐1* and *sus* ^*sext*^ *‐2* were grown on soil in a 16‐h light period under controlled climate conditions. Under these conditions no visible growth differences were observed between *sus* mutants and wild type (Figure 1a). Compromised secondary cell wall biosynthesis leads to thinner and weaker cell walls, often evident as collapsed xylem vessels (Taylor et al., 2000). Therefore, transverse sections of inflorescence stems were prepared to investigate possible cell wall defects in the vascular bundles and the adjacent interfascicular fibers. Light microscopy of the toluidine blue‐stained cross‐sections revealed no anatomical defects in the *sus* mutants (Figure 1b). Deficient primary wall cellulose synthesis interferes with cell expansion visible as reduced hypocotyl elongation in etiolated seedlings (Fagard et al., 2000). To inspect possible defects in primary cell wall biosynthesis we compared the hypocotyl length in etiolated 3‐day‐old wild‐type, *sus* ^*quad*^, *sus* ^*sext*^ *‐1*, and *sus* ^*sext*^ *‐2* seedlings, but also in these experiments no differences between the *sus* mutants and wild type was observed (Figure 1c,d).

**Figure 2:**
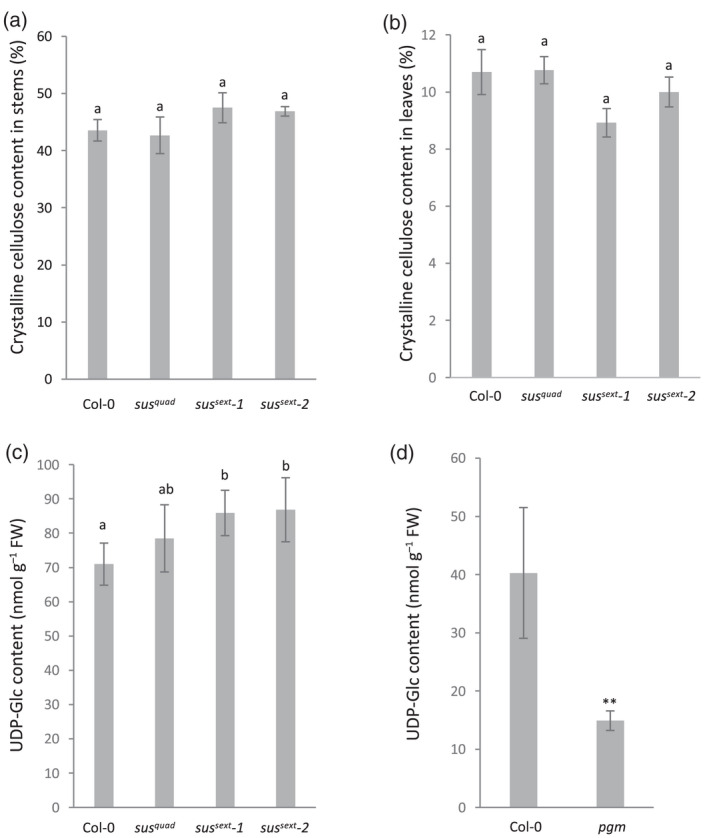
Cellulose and UDP‐glucose content in Arabidopsis *sus* mutants. Crystalline (Updegraff) cellulose content in (a) stems of 10‐week‐old and (b) rosette leaves of Col‐0 and *sus* ^*quad*^, *sus* ^*sext*^ *‐1*, and *sus* ^*sext*^ *‐2*. Values are means ± SE, *n* = 6 biological replicates. (c) UDP‐glucose content in rosette leaves of Col‐0, *sus* ^*quad*^, *sus* ^*sext*^ *‐1*, and *sus* ^*sext*^ *‐2* in the middle of the day. (d) UDP‐glucose content in rosette leaves of Col‐0 and *pgm* at the end of the night. **Student's *t*‐test, *P* < 0.01. Plants were grown in short days (8‐h light, 16‐h dark). Values are means ± SD, *n* = 5 biological replicates. One‐way ANOVA, *post‐hoc* Tukey HSD test. Different letters indicate significant differences (*P* < 0.05). UDP‐Glc, UDP‐glucose.

It is possible that lack of SUS activity leads to more subtle effects on cellulose biosynthesis, which would not manifest as anatomical and growth defects. However, quantification of cellulose content in the stems and rosette leaves of *sus* ^*quad*^, *sus* ^*sext*^ *‐1*, and *sus* ^*sext*^ *‐2* revealed no changes (Figure 2a,b). To elucidate the reason for the lack of growth phenotypes and cellulose defects further we quantified the UDP‐glucose pool in rosette leaves of wild type, *sus* ^*quad*^, *sus* ^*sext*^ *‐1*, and *sus* ^*sext*^ *‐2*. Reliable quantification of UDP‐glucose from plant extracts requires rapid quenching of metabolism, extraction, and precise liquid chromatography–tandem mass spectrometry (LC‐MS/MS) analysis. UDP‐glucose analysis is difficult to perform with success on traditional reversed phase chromatography due to the polarity of the compound. Here we developed a new method combining hydrophilic interaction chromatography (HILIC) with the addition of medronic acid in the mobile phase, which was previously proposed to improve peak shapes of phosphate‐containing compounds (Hsiao et al., 2018). A combination of polymer‐based HILIC column with medronic acid in the mobile phase resulted in improved peak symmetry (Figure S1). Thus, together with MS/MS including carbon‐13 labeled UDP‐glucose as an internal standard the method provides an alternative for accurate determination of UDP‐glucose levels. This analysis revealed that the size of the UDP‐glucose pool in the rosette leaves of *sus* ^*quad*^ was in fact increased by 10.6% and by 21.0% and 22.4% in *sus* ^*sext*^ *‐1* and *sus* ^*sext*^ *‐2*, respectively (Figure 2c). These counter‐intuitive results indicated that alternative UDP‐glucose synthesis pathway(s) were upregulated and compensated for the missing SUS activity. As an additional control, we analyzed rosette leaf extracts of the starchless Arabidopsis *pgm* mutant, which exhibits reduced UDP‐glucose levels compared with wild type at the end of the dark period (Gibon et al., 2006). In line with the previously published results, the UDP‐glucose levels were reduced in *pgm* corroborating the LC‐MS/MS analysis (Figure 2d).

Based on these observations, it can thus be concluded that SUS activity is not required for cellulose biosynthesis in Arabidopsis resolving the long‐standing debate about the role of SUS in cellulose biosynthesis. Sucrose cleavage by invertases followed by hexose phosphorylation by hexokinases and fructokinases, and UDP‐glucose biosynthesis by UGPase activity provide a possible alternative pathway (Barnes & Anderson, 2018; Barratt et al., 2009; Rende et al., 2017). It should be noted, however, that Arabidopsis *sus1sus4* mutants exhibit growth defects under hypoxia pointing to the importance of the SUS pathway under stress conditions (Bieniawska et al., 2007). A role for SUS in plant stress tolerance was recently also supported by the growth defects observed in hybrid aspen *SUSRNAi* lines grown under natural conditions for 5 years (Dominguez et al., 2021).

## EXPERIMENTAL PROCEDURES

### Plant material and growth conditions

The *Arabidopsis thaliana* ecotype Columbia‐0 (Col‐0) was used as the wild‐type control. The quadruple *sus1234* (*sus* ^*quad*^) mutant was described in Barratt et al. (2009). The sextuple mutants *sus12345* ^*1*^ *6* (*sus* ^*sext*^‐1) and *sus12345* ^*2*^ *6* (*sus* ^*sext*^‐2) were generated by CRISPR/Cas9 as described in Fünfgeld et al. (2022). The starchless *pgm* mutant (germplasm CS3092) characterized by Caspar et al. (1985) was obtained from the ABRC (https://abrc.osu.edu/). For the hypocotyl elongation assay, seeds were surface sterilized with 70% ethanol and 0.1% Tween‐20 and sowed on half‐strength Murashige and Skoog plates with 1% sucrose and 0.8% plant agar. Then plates were kept at 4°C for 3 days in the dark followed by light treatment at 22°C for 6 h to ensure uniform germination. After this, the plates were wrapped in aluminum foil and placed vertically at 22°C for 5 days. Images of etiolated seedlings were taken by a Canon EOS 650D camera and analyzed by ImageJ (http://www.imagej.nih.gov/ij/). For the other experiments, plants were grown in soil at 22°C with a photoperiod of 16 h light and 8 h dark (long day) or 8 h light and 16 h dark (short day) and 65% relative humidity. Valoya NS12 LED tubes were used and the light intensity is 150 μmol m^−2^ sec^−1^.

### Anatomy

Sections of the stems from the bottom part of 10‐week‐old wild‐type and mutant plants were stained in 0.1% (w/v) Toluidine blue and rinsed with water for three times. The sections were mounted in water and observed under a Zeiss Axioplan 2 microscope equipped with a Zeiss AxioCam HRc camera. Images were processed and analyzed using ZEN 2 blue edition (Zeiss, Jena, Germany).

### Cellulose analysis

The bottom 5 cm part of stems from 10‐week‐old plants grown in long day conditions were used for cellulose analysis. The freeze‐dried samples were ground in liquid nitrogen with mortar and pestle, and alcohol insoluble residues were extracted by sequentially heating the samples in 80% and 70% ethanol at 95°C for 30 min, followed by treatment with chloroform/methanol (1:1) and washing with 100% acetone. The samples were resuspended in 0.1 m potassium phosphate buffer (pH 7.0) containing 0.01% sodium azide, and starch was removed by digesting the samples twice overnight with α‐amylase (10102814001; 10 U μl^−1^; Roche, Basel, Switzerland) at +37°C, under gentle rotation. Cellulose content was measured using the Updegraff method (Updegraff, 1969) followed by an anthrone assay (Scott & Melvin, 1953) to quantify the released glucose.

### LC‐MS/MS analysis of UDP‐glucose

Rosette leaves of 4‐week‐old wild‐type and *sus* mutants grown in short days were harvested and homogenized in liquid nitrogen. Ten milligrams of each sample was placed into a 1.5 ml Eppendorf tube together with 250 μl of ice‐cold extraction medium (chloroform/methanol, 3:7) and incubated at −20°C for 2 h. After this, 10 μl 50 μm UDP‐α‐d‐[UL‐^13^C6]glucose (Omicron Biochemicals, Inc., South Bend, IN, USA) was added to each sample as an internal standard. Samples were then extracted twice with 200 μl of ice‐cold water, the aqueous layers were combined and dried in a freeze‐dryer. The dried samples were dissolved in 50 μl of 50% methanol and diluted 10‐fold before the analysis by LC‐MS/MS. The separation of metabolites was achieved by injecting 3 μl of a sample to a HILIC column (iHILIC‐(P) Classic, PEEK, 150 × 2.1 mm, 5 μm; HILICON, Umeå, Sweden) and mobile phase composed of (A) 10 mm ammonium acetate in water pH 9.4, and (B) 10 mm ammonium acetate in 90% acetonitrile pH 9.4 at a flow rate of 0.2 ml min^−1^. Ammonium hydroxide was used to adjust pH of the mobile phase and mobile phase was supplemented by 5 μm medronic acid. The gradient elution program was set as follows: 0.0 min (95% B), 15 min (30% B), 18 min (30% B), 19 min (95% B), and 27 min (95% B). The LC‐MS/MS system consisted of an Agilent 1290 UPLC connected to an Agilent 6490 triple quadrupole (Agilent, Santa Clara, CA, USA). Analytes were ionized in electrospray source operated in the negative mode. The source and gas parameters were set as follows: ion spray voltage −3.5 kV, gas temperature 150°C, drying gas flow 11 L min^−1^, nebulizer pressure 20 psi, sheath gas temperature 350°C, sheath gas flow 12 L min^−1^, and fragmentor 380 V. Multiple reaction monitoring transitions of UDP‐glucose and UDP‐glucose‐^13^C_6_ were optimized by using flow injection analysis (Table S1). Quantification of UDP‐glucose was conducted based on calibration curve using UDP‐glucose‐^13^C_6_ as an internal standard. Linearity of the method was determined through a 10‐point and weighted (1/*x*) calibration, which covered the range from 15 nm to 10 μm with the coefficient of determination *R* ^2^ = 0.9958. The limit of detection and limit of quantification were determined as a peak height of UDP‐glucose that is 3‐ and 10‐fold the signal‐to‐noise ratio, respectively. Accuracy was evaluated by spiking the real samples with the standard solution of UDP‐glucose (*n* = 3) at three concentration levels (0.1, 1.5, and 5 μm); accuracy was in an acceptable range of 100 ± 15%. Precision was calculated as RSD of repeated measurements (*n* = 6) of the spiked samples at three concentration levels; precision did not exceed 15%. All validation parameters are listed in Table S2.

## AUTHOR CONTRIBUTIONS

WW and SV carried out experiments and analyzed data, OH and TM developed and performed the UDP‐glucose quantification assay, WW and TN conceived the project, and TN wrote the article with contributions from all the authors.

## CONFLICT OF INTERESTS

The authors declare that they have no competing interests.

## Supporting information


**Table S1** **.** LC‐MS/MS reaction monitoring parameters.
**Table S2.** Validation paramaters for quantification of UDP‐glucose in plant extracts.
**Figure S1.** Example of UDP‐glucose chromatograms.

## Data Availability

All relevant data can be found within the manuscript and its supporting materials.

## References

[tpj15752-bib-0001] Amor, Y. , Haigler, C.H. , Johnson, S. , Wainscott, M. & Delmer, D.P. (1995) A membrane‐associated form of sucrose synthase and its potential role in synthesis of cellulose and callose in plants. Proceedings of the National Academy of Sciences of the United States of America, 92(20), 9353–9357. 7568131 10.1073/pnas.92.20.9353 PMC40983

[tpj15752-bib-0002] Barnes, W.J. & Anderson, C.T. (2018) Cytosolic invertases contribute to cellulose biosynthesis and influence carbon partitioning in seedlings of *Arabidopsis thaliana* . The Plant Journal, 94(6), 956–974. 29569779 10.1111/tpj.13909

[tpj15752-bib-0003] Baroja‐Fernandez, E. , Munoz, F.J. , Li, J. , Bahaji, A. , Almagro, G. , Montero, M. et al. (2012) Sucrose synthase activity in the sus1/sus2/sus3/sus4 Arabidopsis mutant is sufficient to support normal cellulose and starch production. Proceedings of the National Academy of Sciences of the United States of America, 109(1), 321–326. 22184213 10.1073/pnas.1117099109 PMC3252950

[tpj15752-bib-0004] Barratt, D.H. , Derbyshire, P. , Findlay, K. , Pike, M. , Wellner, N. , Lunn, J. et al. (2009) Normal growth of Arabidopsis requires cytosolic invertase but not sucrose synthase. Proceedings of the National Academy of Sciences of the United States of America, 106(31), 13124–13129. 19470642 10.1073/pnas.0900689106 PMC2722301

[tpj15752-bib-0005] Bieniawska, Z. , Paul Barratt, D.H. , Garlick, A.P. , Thole, V. , Kruger, N.J. , Martin, C. et al. (2007) Analysis of the sucrose synthase gene family in Arabidopsis. The Plant Journal, 49(5), 810–828. 17257168 10.1111/j.1365-313X.2006.03011.x

[tpj15752-bib-0006] Caspar, T. , Huber, S.C. & Somerville, C. (1985) Alterations in growth, photosynthesis, and respiration in a starchless mutant of *Arabidopsis thaliana* (L.) deficient in chloroplast phosphoglucomutase activity. Plant Physiology, 79, 11–17. 16664354 10.1104/pp.79.1.11 PMC1074821

[tpj15752-bib-0007] Dominguez, P.G. , Donev, E. , Derba‐Maceluch, M. , Bunder, A. , Hedenstrom, M. , Tomaskova, I. et al. (2021) Sucrose synthase determines carbon allocation in developing wood and alters carbon flow at the whole tree level in aspen. The New Phytologist, 229(1), 186–198. 32491203 10.1111/nph.16721

[tpj15752-bib-0008] Endler, A. & Persson, S. (2011) Cellulose synthases and synthesis in Arabidopsis. Molecular Plant, 4(2), 199–211. 21307367 10.1093/mp/ssq079

[tpj15752-bib-0009] Fagard, M. , Desnos, T. , Desprez, T. , Goubet, F. , Refregier, G. , Mouille, G. et al. (2000) PROCUSTE1 encodes a cellulose synthase required for normal cell elongation specifically in roots and dark‐grown hypocotyls of Arabidopsis. Plant Cell, 12(12), 2409–2424. 11148287 10.1105/tpc.12.12.2409 PMC102227

[tpj15752-bib-0010] Fujii, S. , Hayashi, T. & Mizuno, K. (2010) Sucrose synthase is an integral component of the cellulose synthesis machinery. Plant & Cell Physiology, 51(2), 294–301. 20056592 10.1093/pcp/pcp190

[tpj15752-bib-0011] Fünfgeld, M.M.F.F. , Wang, W. , Ishihara, H. , Arrivault, S. , Feil, R. , Smith, A.M. et al. (2022) Sucrose synthases are not involved in starch synthesis in *Arabidopsis* leaves. Nature Plants. 10.1038/s41477-022-01140-y. PMC9122829 35484201

[tpj15752-bib-0012] Gibon Y , Usadel B , Blaesing OE , Kamlage B , Hoehne M , Trethewey R et al. (2006). Integration of metabolite with transcript and enzyme activity profiling during diurnal cycles in *Arabidopsis* rosettes. Genome Biology 7, R76. Available from: 10.1186/gb-2006-7-8-r76 16916443 PMC1779593

[tpj15752-bib-0013] Guerriero, G. , Fugelstad, J. & Bulone, V. (2010) What do we really know about cellulose biosynthesis in higher plants? Journal of Intergrative Plant Biology, 52(2), 161–175. 10.1111/j.1744-7909.2010.00935.x 20377678

[tpj15752-bib-0014] Haigler, C.H. , Ivanova‐Datcheva, M. , Hogan, P.S. , Salnikov, V.V. , Hwang, S. , Martin, K. et al. (2001) Carbon partitioning to cellulose synthesis. Plant Molecular Biology, 47(1/2), 29–51. 11554477

[tpj15752-bib-0015] Hsiao, J.J. , Potter, O.G. , Chu, T.W. & Yin, H. (2018) Improved LC/MS methods for the analysis of metal‐sensitive analytes using Medronic acid as a Mobile phase additive. Analytical Chemistry, 90(15), 9457–9464. 29976062 10.1021/acs.analchem.8b02100

[tpj15752-bib-0016] McFarlane, H.E. , Doring, A. & Persson, S. (2014) The cell biology of cellulose synthesis. Annual Review of Plant Biology, 65, 69–94. 10.1146/annurev-arplant-050213-040240 24579997

[tpj15752-bib-0017] Rende, U. , Wang, W. , Gandla, M.L. , Jonsson, L.J. & Niittyla, T. (2017) Cytosolic invertase contributes to the supply of substrate for cellulose biosynthesis in developing wood. The New Phytologist, 214(2), 796–807. 28032636 10.1111/nph.14392

[tpj15752-bib-0018] Ruan, Y.L. , Llewellyn, D.J. & Furbank, R.T. (2003) Suppression of sucrose synthase gene expression represses cotton fiber cell initiation, elongation, and seed development. Plant Cell, 15(4), 952–964. 12671090 10.1105/tpc.010108 PMC152341

[tpj15752-bib-0019] Scott, T.A. & Melvin, E.H. (1953) Determination of dextran with Anthrone. Analytical Chemistry, 25(11), 1656–1661.

[tpj15752-bib-0020] Song, D. , Shen, J. & Li, L. (2010) Characterization of cellulose synthase complexes in *Populus* xylem differentiation. The New Phytologist, 187(3), 777–790. 20546138 10.1111/j.1469-8137.2010.03315.x

[tpj15752-bib-0021] Stein, O. & Granot, D. (2019) An overview of sucrose synthases in plants. Frontiers in Plant Science, 10, 95. 30800137 10.3389/fpls.2019.00095 PMC6375876

[tpj15752-bib-0022] Taylor, N.G. , Laurie, S. & Turner, S.R. (2000) Multiple cellulose synthase catalytic subunits are required for cellulose synthesis in Arabidopsis. Plant Cell, 12(12), 2529–2540. 11148295 10.1105/tpc.12.12.2529 PMC102235

[tpj15752-bib-0023] Updegraff, D.M. (1969) Semimicro determination of cellulose inbiological materials. Analytical Biochemistry, 32(3), 420–424. 5361396 10.1016/s0003-2697(69)80009-6

