# Supplementary material for: Sucrose synthase activity is not required for cellulose biosynthesis in Arabidopsis
Source: Plant J. 2022 Apr 21;110(5):1493–7. doi: 10.1111/tpj.15752 (PMC9322421; doi:10.1111/tpj.15752)
Supplement: Supplementary file 1 — Table S1 . LC‐MS/MS reaction monitoring parameters. Table S2. Validation paramaters for quantification of UDP‐glucose in plant extracts. Figure S1. Example of UDP‐glucose chromatograms. [file TPJ-110-1493-s001.docx]

**Table S1.** Multiple reaction monitoring parameters.

| **Analyte** | **Precursor ion** | **Product ion** | **Collision energy [V]** | **Dwell time [ms]** | **Type of transition** |
| --- | --- | --- | --- | --- | --- |
| UDP-Glc | 565.0 | 322.9 | 25 | 150 | quantifier |
|  |  | 78.8 | 77 | 150 | qualifier |
| UDP-Glc-^13^C_6_ | 571.0 | 322.8 | 21 | 150 | quantifier |
|  |  | 78.9 | 77 | 150 | qualifier |


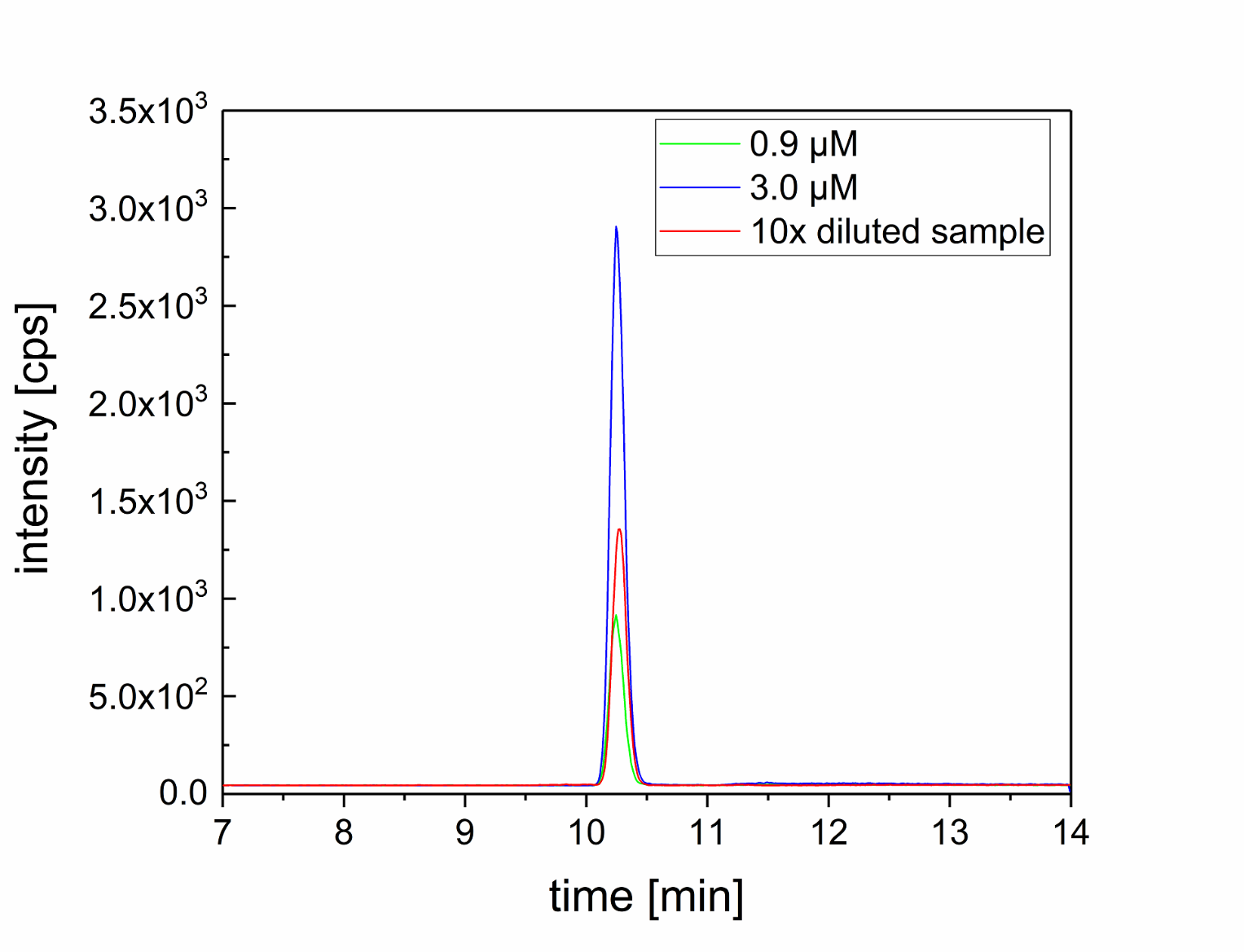


**Figure S1.** Extracted and overlayed chromatograms of UDP-Glucose (MRM transition: 565.0 > 322.9) from calibration solutions and from 10× diluted sample. Chromatographic conditions as described in LC-MS/MS analysis section.

**Table S2.** Validation paramaters for quantification of UDP-glucose in plant extracts.

| Analyte | LOD [nM] | LOQ [nM] | *R*^2^ | Precision [%] | | | Accuracy [%] | | |
| --- | --- | --- | --- | --- | --- | --- | --- | --- | --- |
|  |  |  |  | Low | Medium | High | Low | Medium | High |
| UDP-Glc | 5 | 15 | 0.9958 | 1.1 | 12.6 | 1.1 | 88.8 | 88.6 | 87.6 |
